# Combination of transsphenoidal endoscopic surgery and presurgical somatostatin analogs in thyrotropin (TSH)-secreting pituitary adenomas: Treatment outcome and long-term remission at a single pituitary center

**DOI:** 10.3389/fendo.2022.1061029

**Published:** 2022-11-28

**Authors:** Jie Liu, Yamei Yang, Lian Duan, Xiaofeng Chai, Huijuan Zhu, Kan Deng, Xiaolan Lian, Yong Yao

**Affiliations:** ^1^ Department of Neurosurgery, Peking Union Medical College Hospital, Chinese Academy of Medical Science and Peking Union Medical College, Beijing, China; ^2^ Key Laboratory of Endocrinology of National Health Commission, Beijing, China; ^3^ Department of Endocrinology, Peking Union Medical College Hospital, Chinese Academy of Medical Science and Peking Union Medical College, Beijing, China

**Keywords:** thyrotropin secreting adenoma, somatostatin analog, transphenoidal endoscopic surgery, remission, prediction

## Abstract

**Background:**

Thyrotropin (TSH)-secreting pituitary adenomas (TSHomas) account for an extremely rare group of pituitary adenomas. Few studies examined the sensitivity and efficacy of presurgical somatostatin analogs (SSAs) and described the long-term remission under such treatment modality. The aim of the present study was to assess the efficacy of presurgical SSA treatment and long-term remission after surgery.

**Methods:**

A retrospective cohort of 65 TSHoma patients who received endoscopic transsphenoidal pituitary surgery between 2011 and 2020 in a single pituitary center in China was established. Data were analyzed for sex differences and different types of SSA and ultimately to explore the hormonal cutoff for remission prediction.

**Results:**

TSHomas had a predominant female preference in this cohort (43 women vs. 22 men). Baseline FT3 was higher in men [7.543 ± 2.407 vs. 5.58 (4.99, 6.58), *p* = 0.019], which was consistent with its longer diagnosis time and larger tumor volume. The median medication time for hormonal control was 2. 5 days for short-acting SSA and 4. 0 weeks for long-term SSA. Patients with long-acting SSA had a shrinking maximum tumor diameter at a median of 1.0 (−1.6, 4.925) mm. Only 10 patients (15.38%) were not in complete remission among whom 8 patients were not en-bloc resected and 2 patients had tumor recurrence after 81.6 and 10. 7 months of complete removal. Postsurgical thyroid hormones (within 1 week) of TSH <0.094 μIU/ml were identified as the cutoff for remission using the ROC curve.

**Conclusions:**

The combination of endoscopic transsphenoidal surgery and presurgical SSA TSHomas provided a higher long-term remission for TSHomas.

## 1 Introduction

Thyrotropin (TSH)-secreting pituitary adenomas (TSHomas), which were originally noted as inappropriate secretion of TSH (IST), are characterized by high levels of circulating free thyroid hormones, non-suppressed TSH levels, and pituitary lesions (1). Since the first case of TSHoma reported in 1960 (2), more than 500 cases have been diagnosed with the availability of more sensitive hormonal assays and imaging techniques (3). The prevalence of this disease is around one per million and accounts for 0.5%–3.0% of all pituitary adenomas (4, 5). Most TSHoma patients are diagnosed in the fourth–sixth decade of their lives, with symptoms of hyperthyroidism and no significant gender difference (3). As pit-1 lineage adenomas, TSHomas express somatostatin receptors (SSTRs) including SSTR2, SSTR3, and SSTR5 and can be suppressed by somatostatin analogs (SSAs) consequently. In this way, SSA therapy was also valued in the treatment of TSHomas, and some studies have verified its ability to normalize TSH levels in 90% TSHoma patients who failed in surgical treatment (1). In addition, SSAs are also recommended in the differential diagnosis of TSHoma with resistance of the thyroid hormone (RTH) as an approach for diagnostic treatments (6–8). To date, transsphenoidal surgery is regarded as the first-line therapy for TSHomas, and some studies suggested that up to 80% of patients were able to restore euthyroidism postoperatively (9). An endoscopic technique that enables the surgeon to observe the surgical field clearly and comprehensively is more widely used in pituitary surgeries (10, 11). Considering the rarity of TSHomas, multiple treatment modalities had been applied in clinical practice, and the clinical outcomes of TSHomas using each therapy have not been fully studied, especially the combination of endoscopic surgery and SSA. In this article, we aim to describe a TSHoma case series in a single institution which might be the largest one in recent years, to explore the effectiveness of combination therapy, and to yield a predictive cutoff using postsurgical thyroid function for the complete remission of TSHoma patients.

## 2 Material and methods

### 2.1 Patient inclusion

**Figure 1:**
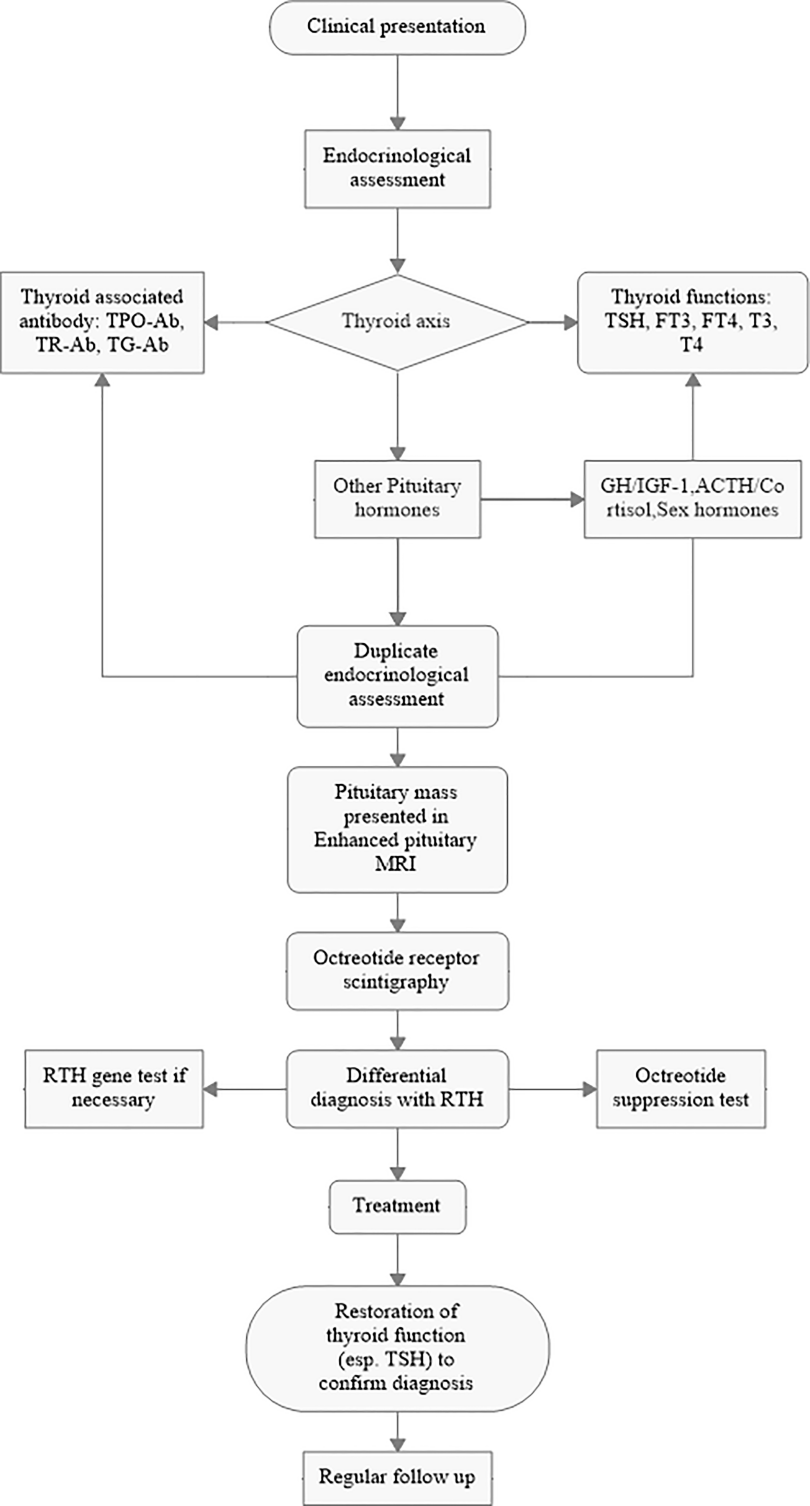
Workflow of diagnosis for TSHomas.

We retrospectively collected 148 consecutive patients from the TSHoma database of Peking Union Medical College Hospital (PUMCH) from 2011 to 2020. The diagnosis criteria were as follows: 1) high levels of circulating free thyroid hormones with non-suppressed TSH levels were repeatedly confirmed and 2) enhanced magnetic resonance imaging (MRI) identified a tumor mass in the sellar region 3) with or without clinical symptoms of hyperthyroidism. The process of diagnosis followed the workflow shown in **Figure 1**.

To investigate the surgical outcome of endoscopic surgery, we excluded patients who underwent microscopic transsphenoidal surgery and craniotomy for pituitary adenoma resection. In these patients, we strictly refined that only 68 patients received endoscopic transsphenoidal pituitary surgery, and all the surgeries were performed by two experienced neurosurgeons (YY and DK). Two of the patients were admitted for the second time for not undergoing en-bloc resection in the first operation in other hospitals that might yield statistical disturbance and one patient had missing data; thus, these patients were excluded in this study. In total, 65 patients were included in this study. The tumors were classified into microadenomas (< 10 mm) and macroadenomas (≥ 10 mm) based on their maximum diameter.

### 2.2 Thyroid function tests

Thyroid functions were performed in the PUMCH laboratory. The levels of serum TSH, FT3, FT4, T3, and T4 were tested using the direct chemiluminescence method (ADVIA Centaur, Siemens, USA). The reference ranges were as follows: TSH 0.38–4. 34 μIU/ml, FT3 1.80–4.10 pg/ml, FT4 0.81-1. 89 ng/dl, T3 0.66–1. 92 ng/ml, and T4 4.30–12.50 μg/dl.

### 2.3 Somatostatin receptor scintigraphy and thyroid sonography

Somatostatin receptor scintigraphy was performed with ^99^mTc-labeled octreotide intravenously administered, and then whole-body scintigraphy was performed 1 and 4 h after injection. The thyroid ultrasound examination was performed using Philips iU22 (8– 15 MHz).

### 2.4 Somatostatin analog treatment

Patients would undergo SSA therapy to control the thyrotoxic symptom and thyroid hormones before surgery if they showed sensitivity to SSA in the octreotide suppression test (OST) and could tolerate SSA treatment according to the recommendation of the European Thyroid Association Guidelines (2013) (1) and the Chinese Expert Consensus (2017). Patients with a suppression ratio of TSH over 50% in OST were empirically defined as “SSA responding” and then would receive presurgical SSA. The medical preference for short-acting or long-acting SSA as presurgical preparation was discussed by the pituitary multidisciplinary team (MDT) of the PUMCH with a thorough evaluation of the general condition, symptom, tumor volume and invasiveness, potential complication, and risk–benefit ratio. After communication with the patient and their relatives, we made a common decision about the SSA type to be used.

### 2.5 Follow-up and remission

We conducted a close follow-up on clinical symptoms, thyroid functions, and pituitary MRI of those patients included in this study. Remission status classified as short-term (3 months) and long-term was documented until May 2022, and the available data were therefore analyzed. For the patients without total thyroidectomy, the complete remission criteria included 1) TSH, FT3, and FT4 within or below the normal ranges for at least 3 months without the use of adjuvant drugs and 2) resolution of neuroradiological lesions after surgery. For patients who were previously thyroidectomized, endocrine studies should show a stable, suppressed TSH status with individualized thyroid hormone substitution, and postsurgical MRI did not show tumor residue. Due to the limitation and flaw of the nature of this retrospective study, not all patients followed the scheduled examination of follow-up, thus resulting in data censorship and potential selection bias in this remission study.

### 2.6 Statistical analysis

Due to the higher incidence rate of TSHomas in female patients, we arbitrarily made a comparative analysis between different genders to explore if there were possibilities of sexual differences regarding clinical features. In addition, the fixed endoscopic surgical procedure and the surgeons provided a great opportunity to minimize bias and error in postsurgical remission.

Data were expressed as mean ± SD and/or as median and interquartile range (25%–75%), as appropriate. Normally distributed variables were compared using the Student’s *t*-test, preceded by Levene’s test to check variance equality. Nominal data were analyzed by the Fisher’s exact test in 2 × 2 contingency tables or by the *χ* ^2^ test. Non-normal variables were analyzed by the Mann–Whitney *U* test. Statistical analysis was carried out by SPSS version 26.0. Values of *p <*0.05 were considered statistically significant. Charts in this article were plotted using GraphPad Prism 8.

## 3 Results

### 3.1 Demographic features

The mean age was 42.4 ± 13. 8 years for diagnosis and 37.7 ± 13.3 years for disease onset. Although female patients were overwhelmingly proportioned (43 women and 22 men, F/M = 1.95), it did not reveal gender differences for onset age and diagnosis age (*p* = 0.695 and *p* = 0.862, respectively). The median duration before diagnosis was 36 months, and male patients were much easier to be delayed (60 vs. 29, *p* = 0.041), which provided sufficient time for tumor growth, and about 68.8% (44/65) of tumors were macroadenomas. There were 82.8% of the patients who showed signs and symptoms of clinical thyrotoxicosis, while only a small proportion of patients presented weight loss (32.8%), vision impairment (26.6%), and headache (23.4%). It was noteworthy that male patients were more likely to have vision impairment (10/22 vs. 7/42, *p* = 0.019). Gonadotropic deficiency was less seen in TSHomas in the admission assessment. Four of 65 patients (6.15%) were concomitant with such type of hypopituitarism and 3 of them were men. The BMI of TSHoma patients was 23.48 ± 3.66 (male: female 24.56 ± 3.05 vs. 22.87 ± 3.87) kg/m^2^. There were 69.2% of the patients who were concomitant with thyroid nodules and 3 of them (all women) were histologically confirmed to have thyroid cancer (all were papillary thyroid carcinomas and two of them had total thyroidectomy before the diagnosis of TSHoma was made). Seven patients turned out to have plurihormonal adenomas, consisting of 4 having GH/TSH, 2 GH/TSH/PRL, and 1 TSH/PRL (**Table 1**).

**Table 1:** Baseline characteristics of the cohort.

	Overall	Male	Female	*p*-value
Gender	65	22	43	
Diagnosis age	42.4 ± 13.8	43.4 ± 15.1	41.9 ± 13.3	0.695
Onset age	37.7 ± 13.3	37.3 ± 13.7	37.9 ± 13.2	0.862
Duration before diagnosis (months)	36 (12, 94)	60 (36, 99)	29 (9, 94)	0.041
Baseline thyroid functions				
TSH	3.596 (2.567, 5.547)	2.899 (2.047, 4.912)	4.15 (2.735, 6.597)	0.127
FT3	6.087 (5.173, 7.645)	7.543 ± 2.407	5.58 (4.99, 6.58)	0.019
FT4	2.156 (1.940, 3.086)	2.694 ± 0.771	2.12 (1.907, 2.67)	0.126
T3	2.010 (1.640, 2.718)	2.304 ± 0.764	1.95 (1.593, 2.598)	0.401
T4	13.7 (11.9, 15.9)	13.71 (11.838, 23.265)	13.41 (11.9, 15.77)	0.810
Clinical thyrotoxicosis (*n* = 64)	53 (82.8%)	16/22 (72.7%)	37/42 (88.1%)	0.166
Vision impairment (*n* = 64)	17 (26.6%)	10/22 (45.5%)	7/42 (16.7%)	0.019
Weight loss (*n* = 64)	21 (32.8%)	9/22 (40.9%)	12/42 (28.6%)	0.318
Headache (*n* = 64)	15 (23.4%)	6/22 (27.3%)	9/42 (21.4%)	0.600
Macroadenoma (*n* = 64)	44 (68.8%)	17/22 (77.3%)	27/42 (64.3%)	0.397
Microadenoma (*n* = 64)	20 (31.3%)	5/22 (22.7%)	15/42 (35.7%)	
Maximum diameter (mm)	14 (7.95, 22)	19.69 ± 11.56	14 (7.5, 18.85)	0.058
Minimum diameter (mm)	11.19 ± 6.41	12.71 ± 7.68	10.2 (5.2, 14.00)	0.313
Knosp classification				
Grade 0	31	10	21	0.439
Grade 1	13	3	10	
Grade 2	11	4	7	
Grade 3	6	2	4	
Grade 4	4	3	1	
Gonadotropic deficiency, *n* (%)	4/65 (6.15%)	3/65 (46.2%)	1/65 (1.54%)	
Corticotropic deficiency, *n* (%)	1/65 (1.54%)	1 (1.54%)	0	
Height (cm)	164 (160, 170.25)	172.36 ± 7.02	163 (157, 165)	<0.001
Weight (kg)	64.21 ± 13.42	73.14 ± 11.32	57 (52, 67)	<0.001
BMI (kg/m^2^)	23.48 ± 3.66	24.56 ± 3.05	22.87 ± 3.87	0.083
Thyroid nodules	45 (69.2%)	15/22 (68.2%)	30/43 (69.8%)	1.000
Thyroid cancer	3/65 (4.6%)	0/22	3/43	0.545

The majority of TSHomas were macroadenomas with maximum (median: 14 mm) and minimum diameters (median: 11.19 mm). However, no sexual differences regarding tumor diameter (*p* = 0.058) and tumor size type (*p* = 0.397) were observed, although men presented a larger proportion of macroadenomas (77.3% vs. 64.3%). Tumors were classified according to the Knosp grading system, where 31 patients were in grade 0, 13 patients in grade 1, 11 patients in grade 2, 6 patients in grade 3, and 4 patients in grade 4. A total of 9 patients had suprasellar involvement presented in MRI.

The basal levels of TSHomas presented in a typical non-suppressed TSH pattern. TSH was at a median of 3.596 μIU/ml which remained in the normal range, whereas FT3, FT4, T3, and T4 were higher than normal ranges, with median levels of 6. 087 pg/ml, 2. 156 ng/dl, 2. 01 ng/ml, and 13.7 μg/dl, respectively. Notably, FT3 was much higher in men [7.543 ± 2.407 vs. 5.58 (4.99, 6.58), *p* = 0.019], which was consistent with its longer diagnosis time and larger tumor volume.

### 3.2 Presurgical SSA

The 65 patients were admitted and endocrinologically assessed prior to endoscopic surgery, and the octreotide suppression test was conducted to test the sensitivity and efficacy of presurgical SSA. Consequently, a total of 56 patients received SSA treatment prior to surgery and no sex difference was observed in terms of SSA use (*p* = 0.054) or SSA medication time (short-acting SSA: *p* = 0.854, long-acting SSA: *p* = 0.462). Patients with presurgical SSA injection manifested adverse effects (AEs) in a gastrointestinal predominance, and no death occurred during the entire process of medication.

The long-acting SSA that was prescribed was octreotide acetate microspheres for injection (Sandostatin LAR, Novartis) for 25 patients (20 mg im, per month) and 1 patient used lanreotide (Somatuline, Ipsen) (40 mg q2w for 12 weeks) with a median medication time of 12 weeks, in which 3 patients had Thyrozol as a subsidiary treatment for symptom alleviation and 1 patient had a medication history of bromocriptine. The short-acting SSA that was used in 30 patients was octreotide (Sandostatin, 0.1mg sc, per 8 h) with a median medication time of 1. 4 weeks. Additionally, one patient used bromocriptine for 3 weeks and added octreotide (Sandostatin) for 5 days before surgery, which was an exceptional case and, hence, was not included in the presurgical SSA treatment analysis.

One female patient with one injection of Sandostatin LAR developed TSH deficiency (low FT4 associated with non-elevated TSH concentrations), who was also concomitant with papillary thyroid carcinoma (biopsied but not operated), and showed no tumor residue in pituitary MRI 12 months after pituitary surgery. She then received radiofrequency ablation of the thyroid after pituitary surgery and maintained euthyroid status with L-T4 substitution.

Fifty-three patients were examined using somatostatin receptor scintigraphy to test the sensitivity of SSA, and 75.47% of these patients were positive for it. No sex difference was observed (*p* = 1.00) (**Table 2**).

**Table 2:** Presurgical medication and somatostatin receptor scintigraphy.

	Overall	Male	Female	*p*-value
Preoperative SSA	56/65	18/22	39/43	0.427
Short-term SSA	30	6	25	0.054
Long-term SSA	26	12	14	
No use of SSA	9	5	4	
Medication time (short-term)/weeks (*n* = 29)	1.4 (1.0, 2.0)	1.35 (0.55, 2.23)	1.4 (1.0, 2.0)	0.854
Medication time (long-term)/weeks (*n* = 26)	12.0 (8.0, 14.13)	12.0 (5.75, 13.13)	12.0 (11.0, 16.0)	0.462
Time for hormonal control (short-term)/days (*n* = 22)	2.5 (2.0, 5.25)	2.0 (1.0, 4.0)	3.0 (2.0, 6.0)	0.249
Time for hormonal control (long-term)/weeks (*n* = 16)	4.0 (1.0, 8.5)	4.0 (1.0, 10.0)	4.0 (1.0, 4.0)	0.536
Somatostatin receptor scintigraphy (+)	40/53	13/17	27/36	1.00

#### 3.2.1 Medication time for SSA

To explore how much time is needed to control thyroid function (TSH, FT3, FT4), we refined the patients with sole SSA as a presurgical preparation and collected the available data, checking the exact days when the thyroid function (TSH, FT3, FT4) dropped to the normal range for both long-acting and short-acting SSA. The short-acting SSA had a median of 2. 5 days to achieve hormonal control, although two patients presented hormonal rebound because of intolerance of the adverse effect of octreotide and five patients were slightly above the normal upper limit before surgery. Long-acting SSA required a median of 4. 0 weeks to control the thyroid function, and three of the patients also were slightly above the normal upper limit before surgery.

#### 3.2.2 Comparison of long-acting and short-acting SSAs

The baseline thyroid hormone levels were not statistically significant between the short-acting and the long-acting SSA groups (TSH, FT4, T3, T4) but not for FT3 [short-acting SSA vs. long-acting SSA: 5.25 (4.87, 6.41) vs. 6.41 (5.69, 8.04), *p* = 0.003].

No statistical difference was observed in TSH and T3 between the long-acting and short-acting SSAs regardless of the hormonal falling proportion or the absolute value, while the FT3, FT4, and T4 descended even more in the long- acting SSA group (**Table 3**).

**Table 3:** Comparison of thyroid hormones that fall between presurgical long-term and short-term SSAs.

Hormone levels	△TSH (%)	△FT3 (%)	△FT4 (%)	△T3 (%)	△T4 (%)
Short-term SSA	2.564 (1.265, 4.142)	2.265 (1.365, 3.165)	0.638 ± 0.579	0.8 (0.529, 1.063)	2.61 (1.24, 4.2)
	81.30% (51.80, 96.24)	43.51% (29.45, 51.32)	27.43% ± 21.53%	44.37% (29.30, 57.46)	20.08% (14.47, 31.82)
Long-term SSA	1.65 (0.701, 3.427)	3.055 (2.56, 4.77)	1.155 ± 0.787	0.893 (0.66, 1.796)	6 (3.43, 8.11)
	63.74% (32.91, 83.83)	51.55% (41.6, 60.03)	42.42% ± 16.33%	47.56% (36.27,61.42)	40.46% ± 18.24%
*p*-value1	0.137	0.016	0.008	0.297	0.007
*p*-value2	0.066	0.021	0.006	0.337	0.007

Valid data number: long-term SSA (TSH, FT3, FT4): 26; short-term SSA (TSH, FT3, FT4): 28; long-term SSA (T3, T4): 25; short-term SSA (T3, T4): 23. Data were analyzed using independent-samples Mann–Whitney U test and independent-samples t-test. p -value1 is for absolute value analysis; p-value 2 is for percentage value analysis.

#### 3.2.3 Changes in tumor size before and after medication

**Figure 2:**
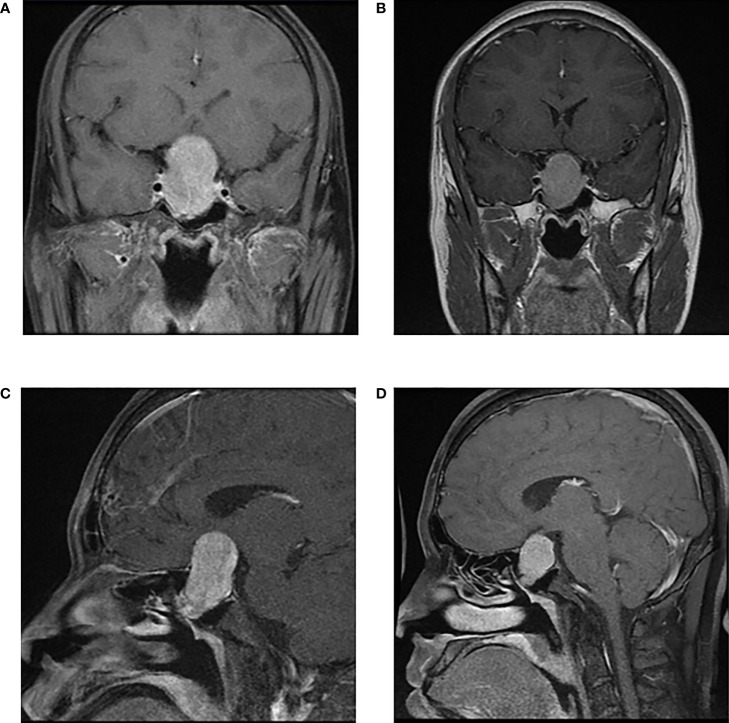
A patient with macroadenoma (46.7×27.7×23.5mm) received long-term SSA (Sandostatin LAR) for 12 weeks and achieved tumor shrinkage (34.2×24.8×20.9mm). Thyroid functions reduced to normal ranges after 4 weeks of medication. However, tumor was in tough texture examined in surgery and rather hard to resect due to long duration of SSA use. | Enhanced Pituitary MRI before SSA: **(A)** axial view and **(C)** sagittal view; Enhanced Pituitary MRI after SSA: **(B)** axial view and **(D)** sagittal view.

In the 26 patients with long- acting SSA, changes in the maximum tumor diameter on MRI before and after medication in a total of 12 patients with available data were compared which showed that long- acting SSA could shrink the maximum tumor diameter at a median of 1.0 (−1.6, 4.925) mm, in which 4 patients (4/12, 33%) had a maximum diameter reduced more than 5 mm, while 2 patients maintained a stable tumor volume although it revealed enlargement (less than 2 mm). A typical MRI image of before and after long-acting SSA use is illustrated in **Figure 2**.

### 3.3 Changes of thyroid functions in the baseline–SSA–surgery process

In the “SSA–surgery” treatment modality, we assessed thyroid functions at baseline, presurgical (after medication), and postsurgical time points. TSH, FT3, T3, and T4 dropped off constantly during the entire process (*p* < 0.05), especially TSH. TSH could descend about 73.11% [3.533 (2.567, 5.547) to 0.95 (0.259, 2.29)] with presurgical SSA use and drop again for 94.95% after surgery [0.95 (0.259, 2.29) to 0.048 (0.016, 0.199)], highlighting the importance of surgical intervention. FT4 also descended significantly after presurgical SSA and surgery, while overall, it kept a relatively stable level at the presurgical SSA to postsurgery levels [2.117 (1.894, 2.599) vs. 1.46 (1.134, 1.714), *p* = 0.608] (**Table 4**).

**Table 4:** Thyroid hormone changes during the baseline–medication–surgery process.

Hormone levels	Baseline levels	Presurgical levels	Postsurgical levels	*p*-value^a^	*p*- value^b^	*p*- value^c^
TSH	3.533 (2.567, 5.547)	0.95 (0.259, 2.29) c	0.048 (0.016, 0.199)	<0.001	<0.001	<0.001
FT3	5.745 (5.148, 7.115)	3.235 (2.67, 3.993) c	2.19 (1.77, 2.72)	<0.001	<0.001	<0.001
FT4	2.117 (1.894, 2.599)	1.394 (1.205, 1.722) c	1.46 (1.134, 1.714)	<0.001	<0.001	0.608
T3	1.963 (1.596, 2.583) b	1.11 (0.86, 1.338) a	0.716 (0.579, 0.908)	<0.001	<0.001	<0.001
T4	13.41 (11.9, 15.595) b	9.83 ± 3.25a	9.166 ± 3.22	<0.001	<0.001	0.048

Valid data number: a = 51, b = 53, and c = 54. Data with skewed distribution were analyzed using the Wilcoxon signed -rank test and data with Gaussian distribution were analyzed using paired-samples t -test.

a Pre- vs. post- SSA.

b Post-SSA vs. postoperation.

c Pre-SSA vs. postoperation.

### 3.4 Remission and predicting factors of remission

The complete remission (CR) rate maintained a generally high level during the entire follow-up. The median follow-up time was 38 months (95% CI: 34.876~41.124) and a total of 10 patients (10/65, 15.38%) were identified as non-CR. The longest follow-up time was 125 months, and 34 patients (52.3%) had been followed up for over 3 years. Two patients were identified to have tumor recurrence with re-elevated thyroid hormones (TSH, FT3, FT4) and reappearance of the tumor in MRI after 81.6 and 10. 7 months of complete removal. All the patients with thyroid cancer in this study were in CR after 9.6, 4.0, and 10. 8 months of follow-up. In the seven plurihormonal TSHoma patients, only two patients were not in CR because of tumor invasion (both were in Knosp grade 4), and the other five patients were in CR (53.1, 3.9, 22.3, 38.4, and 10.8 months of follow-up).

In the non-CR patients, five patients were in Knosp grade 0 and two patients were in Knosp grade 4. The other two patients were in Knosp grades 1 and 2, respectively. Three patients had suprasellar involvement which gave rise to tumor residue and non-CR.

A total of 59 patients received short-acting follow-up (3 months), and the remission rate was 86.44% (51/59). The eight non-CR patients had remnant tumor because of tumor invasion and inaccessibility for en-bloc resection and received further treatment afterward. Apart from the two recurrent patients, all the other patients sustain the CR status during the long-term follow-up.

**Figure 3:**
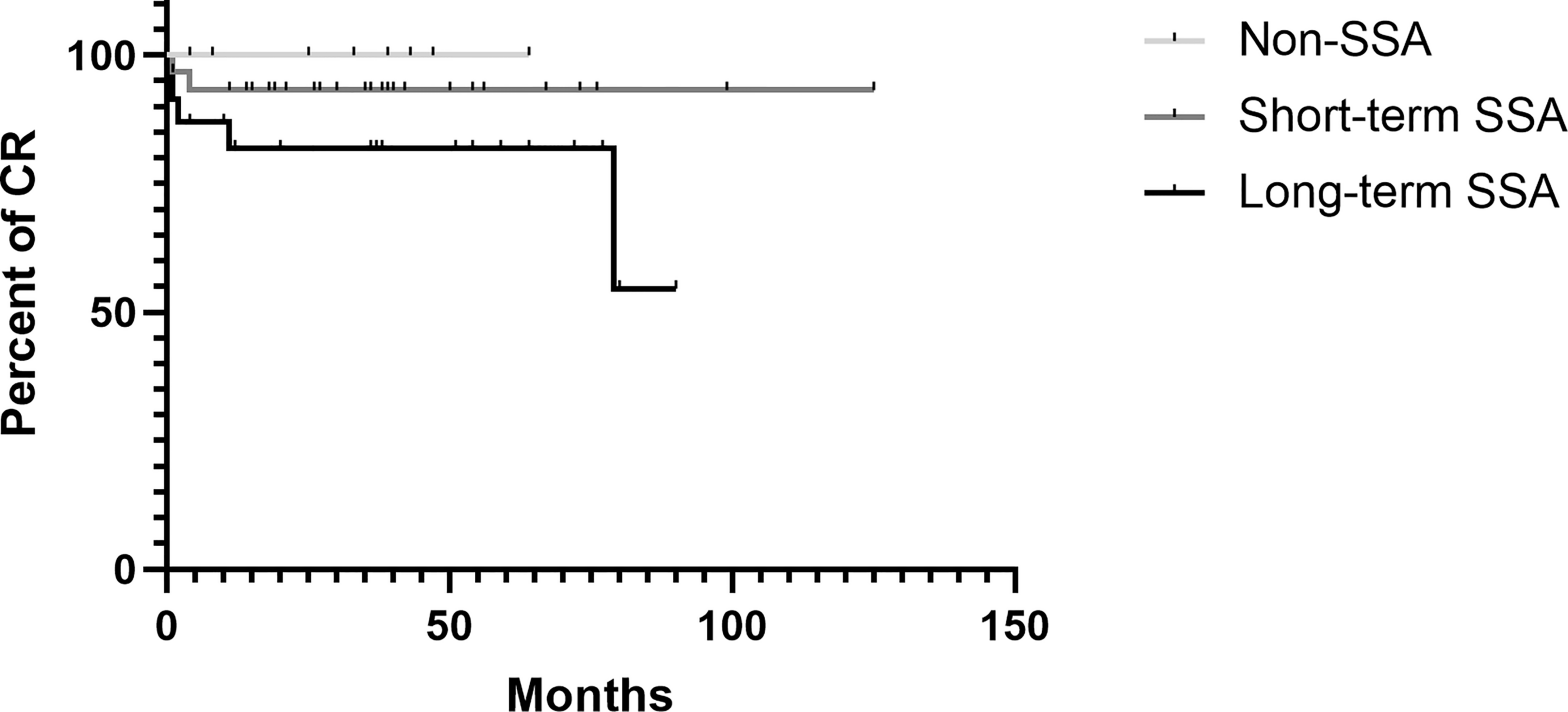
Kaplan Meier estimate illustrating the Cumulative complete remission in the population stratified according to presurgical SSA therapy received.

Only 10 patients (15.38%) were not in complete remission, with 8 of these patients having a remnant tumor and 2 patients having a recurrent tumor after 81.6 and 10. 7 months of complete removal. We made a pairwise comparison of the remission status among the presurgical non-SSA, short-acting SSA, and long-acting SSA groups using the Kaplan–Meier method, and no statistical significance was found (log- rank: non-SSA vs. short-acting SSA, *p* = 0.431; non-SSA vs. long-acting SSA, *p* = 0.097; short-acting SSA vs. long-acting SSA, *p* = 0.024) (Bonferroni- adjusted *p* = 0.0167). Remarkably, long-acting SSAs were preferentially used in patients with more serious symptoms and invasive adenoma (Knosp grade 4: 3/4, 75%) and also had a significantly higher proportion of remnant tumors (7/8, 87.5%) in the non-CR patients. Therefore, the use of long-acting SSA showed its tendency in severe patients and, consequently, a higher rate of non-CR (**Figure 3**).

As for postsurgical complications, 27 patients (41.5%) had immediate postoperative or POD 1–2 polyuria and received one or more doses of ADH, and 6 patients (9.2%) had transient insipidus (prolonged for over 3 days). Two patients (3%) developed permanent insipidus, and they were also diagnosed with panhypopituitarism. When it comes to anterior hypopituitarism, two patients had a simple type of hypopituitarism (one for adrenal insufficiency and one for gonadal insufficiency), and one patient was involved in the three axes of insufficiency (adrenal, gonadal, and thyroid). A total of seven patients (10.8%) developed postsurgical hypopituitarism of the thyroid axis, and four of them were identified in the first 3 months of follow-up and received subsequent levothyroxine substitution. Perioperative CSF leakage occurred in 23 patients (35.4%, available data = 64) and 7 patients developed infection after the operation (10.8%). No significant difference regarding presurgical treatment and remission status was obtained after the analysis.

**Figure 4:**
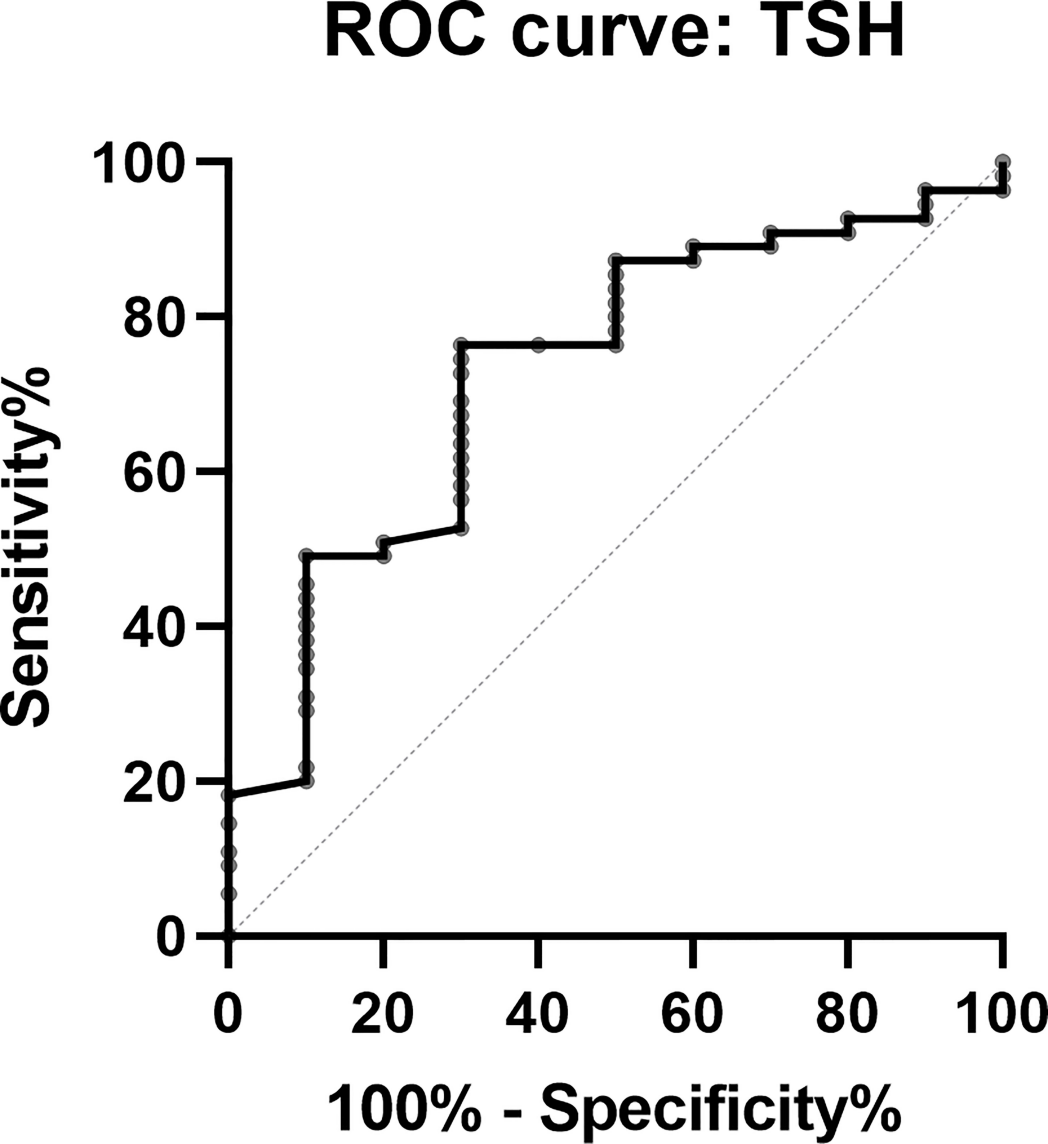
Receiver operating characteristic curve of postsurgical TSH in predicting CR patients.

Despite the fact that postsurgical thyroid function could be influenced by the presurgical use of SSA, we think that postsurgical TSH (within 1 week) could be identified as a predictor on the basis that postsurgical TSH was even lower than the minimal level of TSH under SSA. In this study, TSH maintained a median level of 0.95 (0.259, 2.29) μIU/ml under SSA, and endoscopic surgery enabled it to reach a median level of 0.048 (0.016, 0.199) μIU/ml. Therefore, an ROC curve using the postsurgical TSH was plotted to explore the optimal cutoff for predicting the patients’ remission status, with TSH <0. 094 μIU/ml identified as the alerting cutoff for complete remission (sensitivity 70%, specificity 76.4%) (**Figure 4**). Other factors including postsurgical FT3, FT4, and T4 also presented their predictive values, and the detailed data are presented in **Table 5**.

**Table 5:** Cutoffs of postsurgical thyroid hormones (within 1 week) to predict complete remission.

	Cutoff	Sensitivity	Specificity	AUC	95% CI of AUC	*p*-value
TSH	0.094	0.700	0.764	0.729	0.564–0.894	0.022
FT3	2.065	0.900	0.473	0.709	0.547–0.872	0.037
FT4	1.687	0.700	0.782	0.753	0.607–0.899	0.011
T3	0.7515	0.800	0.655	0.69	0.539–0.841	0.057
T4	12.10	0.600	0.855	0.712	0.535–0.889	0.034

### 3.5 Histological findings

Forty-two patients were immunostained positive for TSH, and women had a higher positivity rate (76.2% vs. 23.8%, *p* = 0.021). To our surprise, 23 patients (35.4%) were immunostained negative for TSH, and they achieved thyroid hormone plunge after surgery and remission. It might be due to inappropriate tissue sampling and technical failure. Considering the great number of TSH-negative patients, there must be other mechanisms responsible for this phenomenon. One should consider that this group of tumors might be part of immature pit1-lineage tumors (2022 WHO Classification of Pituitary Tumors) with focal or variable staining for no hormones or one or more of GH, PRL, and TSH. However, the transcription factors of *pit-1*, *tpit*, *sf-1*, and SSTRs were not stained before the WHO classification was published, which made it hard to verify the hypothesis. Although there were plurihormonal adenomas in this cohort (7/65, 10.8%), pure adenoma still was in the majority, and no sex difference was analyzed (men: women 95.3% vs. 72.7%, *p* = 0.072). As for the Ki-67 index, 28.1% and 56.3% of TSHoma patients were <1% and 1%, which indicated a lower proliferation activity of TSHomas, and no sex difference was observed (*p* = 0.439). The TSHomas also expressed other hormonal markers, with GH expressed the most (78.5%), followed by PRL (44.6%), and p53 expressed the least (12.5%) (**Table 6**).

**Table 6:** Histological findings.

	Overall	Male	Female	*p*-value
TSH (+) in IHC	42 (64.6%)	10/42 (23.8%)	32/42 (76.2%)	0.021
Pure TSH adenoma	58 (89.2%)	17/22 (77.3%)	41/43 (95.3%)	0.072
Plurihormonal adenoma	7 (10.8%)	5/22 (22.7%)	2/43 (4.7%)	
Ki-67 (*n* = 64)				
<1%	18 (28.1%)	4/21 (19.0%)	14/43 (32.6%)	0.429
1%	36 (56.3%)	14/21 (26.4%)	22/43 (51.2%)	
2%	6 (9.4%)	1/21 (4.8%)	5/43 (11.6%)	
3%	4 (6.3%)	2/21 (9.5%)	2/43 (4.7%)	
GH	51 (78.5%)	19/22 (86.4%)	32/43 (74.4%)	0.268
PRL	29 (44.6%)	8/22 (36.4%)	21/43 (48.8%)	0.338
P53 (*n* = 63)	6 (9.5%)	3/20 (15%)	3/43 (6.98%)	1.000

## 4 Discussion

TSHomas are rare functional pituitary adenomas, only accounting for 0.5%–3% of pituitary adenomas. The difficulties in identification diagnosis are derived from the low incidence of TSHomas, insufficient diagnostic experience, and lack of reliable diagnostic tests. Our team has reported case studies of adolescent-onset (12), ectopic (13), and co-secreting TSHomas (14), while in this study, we collected and described the TSHoma case series in a large single center, which was treated and followed up by an experienced pituitary MDT. The mean diagnosis age was 42.4 ± 13. 8 years, and the sex ratio was 1.95:1 (female: male) in our series, which is consistent with the result of female predominance (sex ratio = 1.07) in the latest published meta-analysis (3). Therefore, the possible clinical sex difference was explored in this study. We found that men had longer diagnosis delay, slightly larger tumors, higher baseline FT3 levels, and more occurrence of vision impairment accordingly. We speculate that men tended to present less or were not aware of clinical symptoms and thus caused diagnosis delay. With sufficient time for tumor growth, FT3 was higher and the tumor was larger which might contribute to the involvement of the optic nerve and vision impairment. In GHomas, another pit-1 linkage adenoma, male patients had a worse prognosis (15). We also found that male patients had a larger proportion of non-CR (male: female ratio = 7:3) in TSHomas. However, we still had not confirmed whether the male sex is a risk factor for prognosis in multivariable analysis (data not shown), and it is expected to be fully explored in future studies.

There were 82.8% of patients who presented with signs and symptoms of thyrotoxicosis at their diagnosis, and 68.8% showed pituitary macroadenomas, which was smaller than the 70%–90% macroadenoma ratio concluded before (16, 17) and might result from more sensitive imaging technologies. There were about 16% and 10% of TSHoma patients with hypersecretion of GH or PRL (4), which was also presented in our series. Moreover, plurihormonal TSH adenomas were found to be associated with poorer prognosis in several studies (14, 18), whereas the plurihormonal patients in our cohort maintained a high remission rate (5/7, 71.4%), which might indicate that remission was more associated with tumor invasion. As for complications, 7.7% of the patients in our cohort were diagnosed to have thyroid carcinoma, which was much more than the incidence in the general population (4.2–87.4 per 100,000 persons) (19) and even more than the 4.8% (3/62) that Perticone et al. concluded in TSHoma patients in 2015 (20). As pointed out by Safi et al. (21, 22), a higher TSH level was a risk factor for thyroid cancer, and there was an unusual association between thyrotropic hypersecretion and thyroid carcinogenesis. Frequent thyroid ultrasound examinations in TSHoma patients could also increase the detection rate of thyroid cancers (3).

The presurgical SSA intervention made great effects by descending a median of 73.11% TSH levels. There was no difference in the decline rates of TSH between the long-acting SSA group and the short-acting SSA group. We found that there were no significant changes in the maximum tumor diameters in 12 patients with long-acting SSA before and after medication, with only 33.3% of the patients having a reduced tumor diameter of more than 5 mm. Moreover, long-acting SSA can induce TSH deficiency in TSHomas, although the frequency was relatively low (23). Our surgical experience revealed that in patients with long-acting SSA, tumor texture was rather tougher than in patients with short-acting SSA and that en-bloc resection was difficult to perform. Some of the tumors had to be remnant despite presurgical SSA use. Therefore, our team recommends the short-time use of SSA as long as clinical symptoms and thyroid functions are controlled.

The AEs of SSA were consistent with those in GH -secreting adenoma (24), and most AEs were transient and of mild-to-moderate intensity (24, 25). Preoperative SSA therapy is safe and could also prevent the occurrence of thyroid storm, which was reported in two cases whose TSH and (or) FT4 levels were uncontrolled preoperatively (26, 27).

Endoscopic transsphenoidal pituitary surgery has been introduced in TSHoma for several decades. There is an increasing trend toward endoscopic surgery for pituitary adenoma resection especially its use for larger tumors compared with microsurgery and craniotomy (28). It provided a better surgical view for improved resection (10, 11). However, the remission rate of endoscopic surgery has not been reported in TSHomas.

There was no difference between the 65 patients who underwent endoscopic surgery and the patients of non- endoscopic surgery in our database in terms of baseline characteristics. The remission rate generally remained at a high level during the follow-up. The overall remission rate was 84.61% (55/65), far more than the 69.7% (95% CI 61.1–78.4) or 80% postsurgical biochemical remission rate summarized before (9, 29). However, we still stress the importance of close long-term follow-up for TSHoma patients to avoid tumor recurrence, despite the sensitive detection of non-CR in the short-time follow-up of 3 months. Furthermore, we noticed that TSH could decline from a baseline level of 3.533 (2.567, 5.547) to 0.95 (0.259, 2.29) μIU/ml after SSA use. Remarkably, endoscopic surgery could make TSH reach another huge decrease by about 94.95% for 0.048 (0.016, 0.199) μIU/ml. Based on the surgery effect that TSH dropped far lower than at the presurgical level, we constructed a prediction model for the remission of TSHomas using the postsurgical TSH levels (within 1 week), in which if TSH was less than 0. 094 μIU/ml, patients were highly likely to be CR with a sensitivity of 70% and a specificity of 76.4%. Other postsurgical hormones (within 1 week) including FT3, FT4, and F4 also demonstrated great predictive value for remission which could aid in remission prediction.

We tried to make a univariable and multivariable analysis to identify what factors would impact remission. However, due to the small size and high remission rate of this study, no significant factors were identified to relate closely to remission including maximum tumor diameter, cavernous sinus invasion, Knosp classification, and suprasellar involvement. It is expected that these will be explored in future studies involving a larger sample size.

Radiotherapy is mainly restricted to patients who could not tolerate surgery and is given as adjunctive therapy postoperatively when the residual tumor is present on MRI (30). It was performed in 15.1% of TSHoma cases according to a structured review of 535 adult cases (3). Malchiodi et al. reported a multicenter TSHoma case series with 19 patients treated with radiation therapy (32% for fractioned conventional radiotherapy and 68% for radiosurgery): 31.5% of the patients showed significant tumor reduction and 52% remained unchanged in tumor size. There were 21% of the patients who had normalized thyroid functions between 12 and 24 months after radiation, and 16% developed hypothyroidism. There were no differences in hormonal control and the onset of new pituitary deficiencies regarding radiotherapy type (31). Mouslech et al. systematically reviewed 15 cases of TSHomas treated by gamma -knife radiosurgery and found that 12 patients (92.3%, available data = 13) achieved euthyroid and one hypothyroid (7.6%), while only four patients reported tumor reduction or disappearance (36%, available data = 11) (32). It is therefore speculated that radiotherapy presented a promising therapeutic effect in TSHoma treatment.

As for IHC, we were surprised to find that there were 23 patients negative for TSH staining and all of them showed clinical and biochemical improvement postoperatively. We think that this might be attributed to inappropriate tissue sampling and technical failure, while after a repeat review of pathology, we are more likely to be convinced that there must be other mechanisms regarding hormonal secretion, synthesis, and distribution pattern responsible for this phenomenon. Also, this group of tumors might belong to a specific cell lineage of TSHomas according to the 2022 WHO classification. A similar absence of GH in IHC was also presented in some cases of GHomas and demonstrated that GH immunostaining may not be sufficient for the diagnosis of acromegaly (33). We supposed that the same applies to TSHomas.

There are still some limitations in this study. As a single-center retrospective research, there are inevitably selection bias, missing data, and loss of follow-up, which together with the rarity of TSHomas resulted in difficulties to establish more effective predictive models for remission. Moreover, there are still no consentaneous diagnostic and remission criteria for TSHomas, although we have adopted rigorous criteria for their diagnosis and remission in this study. We are looking forward to exploring the novel risk factors of TSHoma relapse and remission when the sample size is expanded.

## 5 Conclusions

The combination of endoscopic transsphenoidal surgery and presurgical SSA therapy is safe and effective to attain a long-term remission for TSHoma patients and is still the cornerstone for TSHoma treatment. In addition, we presented a new TSHoma case series and established a predictive model for the complete remission of TSHoma, in which TSH <0. 094 μIU/ml (within 1 week after surgery) was the most important predictor.

## Data availability statement

The datasets generated during and/or analyzed during the current study are not publicly available but are available from the corresponding author on reasonable request.

## Ethics statement

The studies involving human participants were reviewed and approved by Ethics Committee of Peking Union Medical College Hospital.

## Author contributions

JL, writing—original draft (lead), review and editing (equal), and formal analysis (lead). YMY, writing —original draft (supporting), review and editing (equal), and validation. LD, methodology (lead). XC, methodology (supporting). HZ, supervision and methodology (supporting). KD, conceptualization (lead), supervision, and resources. XL and YY, conceptualization (supporting) and resources. All authors contributed to the article and approved the submitted version.

## Funding

This work was supported by a grant from the National High Level Hospital Clinical Research Funding(2022-PUMCH-B-114).

## Acknowledgments

The authors would like to express their gratitude to their colleagues at the pituitary multidisciplinary team (MDT) of PUMCH for their careful treatment of the patients and helpful guidance and discussions on this work.

## Conflict of interest

The authors declare that the research was conducted in the absence of any commercial or financial relationships that could be construed as a potential conflict of interest.

## Publisher’s note

All claims expressed in this article are solely those of the authors and do not necessarily represent those of their affiliated organizations, or those of the publisher, the editors and the reviewers. Any product that may be evaluated in this article, or claim that may be made by its manufacturer, is not guaranteed or endorsed by the publisher.
